# Self‐Healing Behavior of Metallopolymers in Complex3D‐Structures Obtained by DLP‐Based 3D‐Printing

**DOI:** 10.1002/chem.202404267

**Published:** 2025-02-12

**Authors:** Michael Klein, Patrick Fesser, Stefan Zechel, Martin D. Hager, Ulrich S. Schubert

**Affiliations:** ^1^ Laboratory of Organic and Macromolecular Chemistry (IOMC) Friedrich Schiller University Jena Humboldtstr. 10 07743 Jena Germany; ^2^ Jena Center for Soft Matter (JCSM) Friedrich Schiller University Jena Philosophenweg 7 07743 Jena Germany; ^3^ Helmholtz-Institute for Polymers in Energy Applications Jena (HIPOLE Jena) Lessingstr. 12-14 07443 Jena Germany; ^4^ Helmholtz-Zentrum Berlin (HZB) Hahn-Meitner-Platz 1 14109 Berlin Germany

**Keywords:** 3D-printing, green chemistry, metallopolymers, polymers, supramolecular chemistry

## Abstract

This current study focusses on the investigation of the self‐healing abilities of metallopolymers containing different kinds of metal complexes, which were processed by direct digital light processing (DLP) based three‐dimensional (3D) printing. For this purpose, 2‐phenoxyethyl acrylate is mixed with ligand‐containing monomers either based on triphenylmethyl(trt)‐histidine or terpyridine, respectively. Either zinc(II) or nickel(II) salts are successfully applied for a complexation of the ligand monomers in solution and, subsequently, photopolymerization is performed. The thermo‐mechanical properties of the obtained metallopolymers were characterized by differential scanning calorimetry (DSC), thermogravimetric analysis (TGA) as well as dynamic mechanical thermal analysis (DMTA). Multiple damages with defined forces ranging from 20 to 1500 mN were introduced into the 3D‐structures and successfully healed within 24 h at 70 °C or 120 °C, respectively without losing the structural integrity of the overall 3D‐structures. Herein, excellent healing efficiencies up to 97 % were determined. Consequently, these hollow structures not only feature very good self‐healing abilities but also excellent retention of the 3D‐structure at and above the healing temperature.

## Graphical abstract

This study presents the digital light processing (DLP) based three‐dimensional (3D) printing of metallopolymers featuring self‐healing abilities. The obtained structures were able to heal mechanical damage with excellent efficiencies up to 97 % without losing their 3D‐structure. Additionally, the healing behavior in dependency of the molecular structure was analyzed in detail revealing new structure‐property‐relationships.

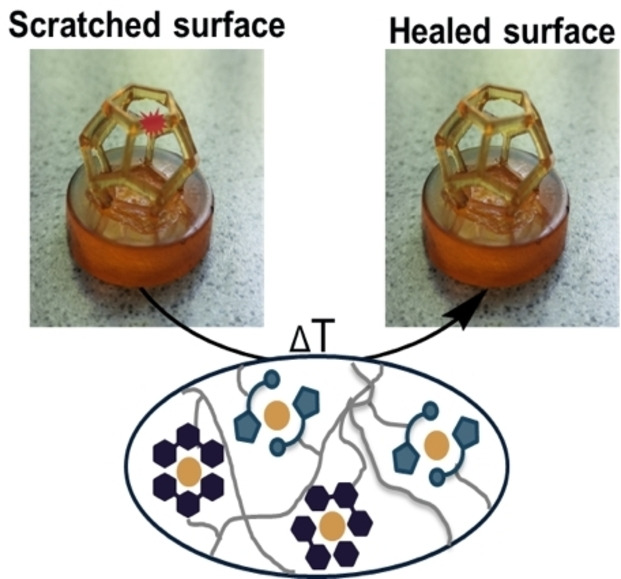

## Introduction

In nature, all biological organisms are able to self‐heal small injuries, like small cuts or bruises, often achieving complete restoration of the damaged sites and of the respective structure.^[1]^ Synthetic materials often lack these features leading to operation failure when damage is introduced.^[2]^ Material engineering research typically focuses on increasing the robustness of new materials to prevent damage from occurring.^[3]^ Nonetheless, man‐made materials reach their limit of reliability and need to be replaced after a certain time.^[4]^ In an attempt to mimic nature, self‐healing materials emerged as a promising class of materials enabling the (partial) recovery of their original performance/properties.^[5]^ This recovery can extend the lifetime of the materials, which reduces replacement costs and increases the sustainability due to less material demand.^[6]^ Due to these advantages, self‐healing materials can be utilized in various applications such as protective films in electronic devices^[7]^ as well as the automotive industry,^[8]^ radiation shields in aerospace,^[9]^ packaging in the food industry^[10]^ or for drug delivery^[11]^ and tissue regeneration^[12]^ in medicine. Many different external triggers like temperature,^[13]^ light,^[14]^ the damage itself,^[15]^ pressure,^[16]^ or electricity^[17]^ can be used in principle to initiate the self‐healing process.

In general, self‐healing can occur *via* an intrinsic or extrinsic pathway.^[18]^ Extrinsic self‐healing relies on the incorporation of healing agent(s) into a polymer matrix.^[19]^ The healing agent is introduced in form of capsules^[15]^ or vascular networks,^[20]^ which contain polymerizable monomers or crosslinkers.^[21]^ Additionally, a catalyst is often dispersed throughout the polymer matrix. When the material is damaged, the shell of the capsule or tube is cracked as well, and the healing agent flows into the damaged site. Upon coming into contact with the catalyst *in situ* polymerization is initiated. This irreversibly converts the healing agent resulting in a singular healing application per damaged site.^[22]^ Further improvements result in extrinsic healable materials based on systems without catalysts^[23]^ or the utilization of solvents as healing agents.^[24]^ Finally, the vascular system can also be connected to external pumps increasing the number of healing cycles.^[25]^

In contrast, intrinsic self‐healing materials enable multiple healing cycles by utilizing reversible covalent bonds^[26]^ or supramolecular interactions.^[27]^ For dynamic covalent bonds several examples are reported in literature like the thermally reversible Diels‐Alder reaction,[^28^, ^29^] the metathesis reaction of disulfide groups,[^30^, ^31^] or the water‐triggered boronic ester exchange reaction.[^32^, ^33^] Self‐healable supramolecular materials can be based on host‐guest interactions,[^34^, ^35^] reversible metal complexes,[^36^, ^37^] π‐π stacking,[^38^, ^39^] ionic interactions,[^40^, ^41^] or hydrogen bonds.[^42^, ^43^] In particular, the usage of metal‐ligand interactions has been of great interested and was researched extensively in the last years.[^13^, ^44^, ^45^, ^46^, ^47^]

The main advantage when using supramolecular interactions, and, in particular, metallopolymers, is the easy tunability of the binding strength of the reversible interaction.^[48]^ This strength can be utilized to adjust the properties of the materials in terms of healability as well as regarding the mechanical performance.^[49]^ However, the application of self‐healing metallopolymers mostly focused on coating applications and the processing was rather simple by solution processing. However, a better processing ability would enable more applications and a broader utilization of such polymers. For this purpose, three‐dimensional (3D) printing can be applied to tailor the materials even further towards desired applications.

One certain technique, which is often applied and also utilized in the current study, is digital light processing (DLP) printing since the utilization of liquid crystal displays enables high resolutions of the printed polymers.[^50^, ^51^] In this context, self‐healing polymers could already be printed by DLP featuring dynamic imine bonds,^[52]^ Diels‐Alder units,^[53]^ polycaprolactones,^[54]^ ionic^[55]^ or hydrogen bonds.^[56]^

However, to the best of our knowledge, there are no reports on the direct 3D‐printing of self‐healable metallopolymers. In our previous work it was already shown that it is possible to obtain smart materials based on dynamic metal‐ligand interactions by DLP‐based 3D‐printing.^[57]^ In the current study, we want to go one step further and are aiming for the investigation of the healability of the metallopolymers in dependency of the chemical structure. Furthermore, the question arises, if the 3D‐printed structure retains after the healing process or if the structure is lost limiting potential applications.

## Results and Discussion

### Monomer Selection

**Scheme 1:**
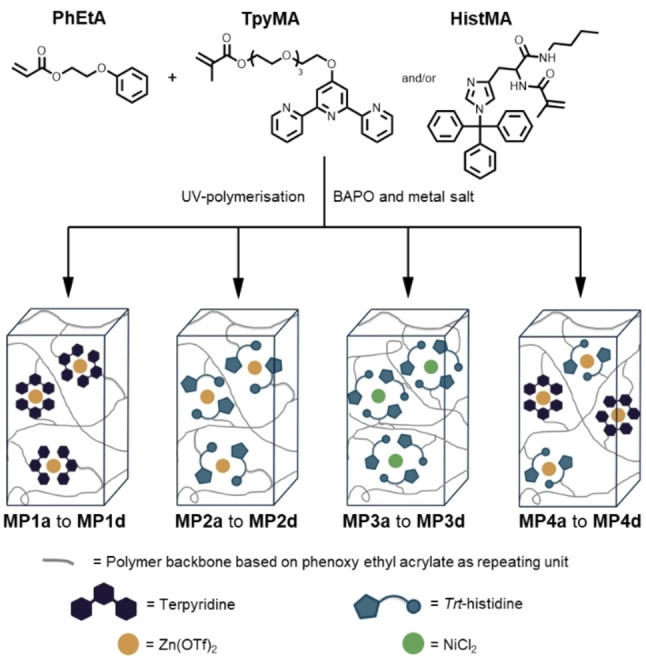
Schematic representation of all 3D‐printed metallopolymers **MP1a** to **MP4d**.

The aim of this study is to obtain supramolecular crosslinked polymers featuring self‐healing abilities by utilizing the direct digital light processing (DLP)‐based three‐dimensional (3D) printing method. In prior studies ligand containing monomers based on 2,2’:6’,2’’‐terpyridine and *N* ^τ^‐tritylhistidine were applied to obtain polymer networks with the ability to self‐heal introduced damages in a very efficient manner in thin film coatings.[^13^, ^58^, ^59^] Due to these findings the ligand‐based monomers *N* ^α^‐methacryloyl‐*N* ^τ^‐tritylhistidine butyl amide (**HistMA**) and 6‐(2,2′:6′,2′′‐terpyridin‐4′‐yloxy)‐tetraethylene glycol methacrylate (**TpyMA**) were synthesized according to literature procedures (see **Scheme** 1 for the structures and **Schemes S1** and **S2** for the synthetic procedure) to apply these ligands systems for the design of metallopolymers.[^57^, ^59^]

Since the 3D‐printing process is performed starting with a solution of the different comonomers, it is required to obtain a solution with dissolved metal complex monomers inside. Therefore, 2‐phenoxy ethyl acrylate (**PhEtA**) was chosen as the comonomer because of its high polarity enabling a high solubility and, thus, a high content of the metal complexes. Other highly polar commercially available monomers feature functional groups like hydroxy or amines, which could potentially interfere with the metal complex formation.

### DLP‐Based 3D‐Printing of Metallopolymer Networks

DLP‐based 3D‐printing is a suitable strategy for the preparation of metallopolymer networks since supramolecular crosslinked polymers can be obtained in defined 3D‐structures. Consequently, we choose this method for the processing of the metallopolymers. The printing conditions including the slice settings as well as the respective amounts for all printed metallopolymers can be found in the Supporting Information (see **Tables S1** and **S2**). The formation of the metallopolymers during the printing process could already be confirmed during a previous study, from which we adopted the processing parameters.^[57]^ In total, four different metallopolymer networks were prepared and a general overview is shown in **Scheme** 1.

Hereby, zinc(II) trifluoromethanesulfonate (Zn(OTf)_2_) was utilized as metal salt (**MP1** for terpyridine as ligand and **MP2** for histidine as ligand). A metallopolymer based on nickel(II) complexes was printed containing histidine as ligand (**MP3**) as well. Lastly, a supramolecular network containing both terpyridine and histidine zinc(II) complexes was obtained by mixing both metal complexes in one printing solution (**MP4**). The herein used metal to ligand ratios were already previously determined by isothermal titration calorimetry (ITC).^[47]^

**Figure 1:**
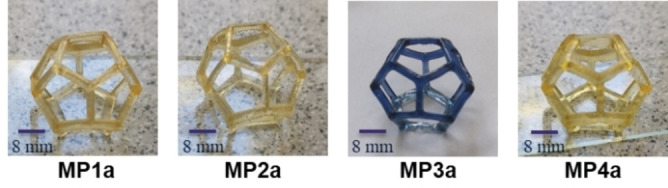
Pictures of the 3D‐printed metallopolymers.

As a 3D‐structure, a dodecahedron was chosen, since it cannot be processed easily with other common processing methods (see **Figure S1** for the model). All samples were printed in form (**Figure** 1) with a crosslinking degree of 5 %, since such an amount of crosslinker can enable self‐healing behavior.^[13]^ For the preparation of metallopolymers the monomer **PhEtA** and the ligand monomer were mixed with the metal salts and hereafter the photoinitiator phenyl‐*bis*(2,4,6‐trimethyl‐benzoyl) phosphine oxide (BAPO) was added.

In case of **MP1**, **TpyMA** and zinc(II) trifluoromethanesulfonate (Zn(OTf)_2_) were utilized and the obtained 3D‐printed hollow structure was free‐standing, featuring a high resolution and clear separation of the struts (**Figure S2**).

The hollow structure obtained by changing the **TpyMA** for **HistMA** (**MP2**) showed the same high resolution and sharp separation (**Figure S3**) but was slightly softer compared to **MP1** after curing. Switching the metal salt to NiCl_2_ and mixing with **PhEtA** as well as **HistMA** resulted in metallopolymer **MP3** after 3D‐printing. This structure was also free‐standing and featured a high resolution (**Figure S4**). Since the combination of **TpyMA** with NiCl_2_ resulted in 3D‐printed polymers without defined structure in a previous study, this combination was not included into this study.^[57]^

Because the DLP‐based 3D‐printing of terpyridine‐zinc(II) and histidine‐zinc(II) complexes respectively resulted in free‐standing dodecahedrons with high resolution an additional hollow structure was printed featuring a 1 to 1 ratio of terpyridine‐zinc(II) and histidine‐zinc(II) complexes (**MP4**). The combination of both complexes caused no loss in resolution while not changing the overall crosslinking degree of the polymer network (**Figure S5**).

The dodecahedrons were printed three times for each metallopolymer (**MPX a**, **MPX b** and **MPX c**) using the same resins and printing settings to confirm the reproducibility of this method. Additionally, this enables the usage of an individual dodecahedron for each experiment (thermal characterization, thermal stability and self‐healing investigation) since these investigations can result in thermal or mechanical damaging and, thus, a loss of the hollow structures.

At last, the metallopolymers were printed in form of rods using the corresponding 3D‐model (**MPX d**, see **Figure S1**). This geometry is required for the dynamic mechanical thermal analysis (DMTA), which will be discussed in the upcoming section of the manuscript.

### Thermomechanical Characterization

The characterization of the thermal and mechanical properties is a key step when investigating the self‐healing ability of 3D‐printed metallopolymers. Therefore, thermogravimetric analysis (TGA), differential scanning calorimetry (DSC) as well as dynamic mechanical thermal analysis (DMTA) were performed for all DLP‐based 3D‐printed metallopolymers obtained by using the dodecahedrons (TGA and DSC, see **Figure S1**) or the rod shapes (DMTA). For the investigation of TGA and DSC, the metallopolymers **MPX a** were utilized, and the results are summarized in **Table** 1. The respective TGA‐ and DSC‐curves are depicted in the Supporting Information (**Figures S6** to **S13**).

**Table 1:** Summary of the investigated thermal properties (glass transition temperature *T*_g_ *via* differential scanning calorimetry (DSC) or dynamic mechanical thermal analysis (DMTA) and degradation temperature *T*_d_ *via* thermogravimetric analysis (TGA)).

	**MP1 a**	**MP2 a**	**MP3 a**	**MP4 a**
*T*_g_^a^ (DSC) [°C]	12	16	8	15
*T*_g_^b^ (DMTA) [°C]	40	29	65	42
*T_d_* ^c^ [°C]	289	244	225	245

^a)^ 3 ^rd^ heating cycle, heating rate: 10 K min^−1^; ^b)^ heating rate: 2 K min^−1^, ^c)^ heating rate: 10 K min^−1^, nitrogen atmosphere.

The analysis of the degradation temperature (*T*_d_) *via* TGA revealed a high thermal stability above 220 °C for all metallopolymers. This enables high healing temperatures without damaging the molecular polymer structure. Furthermore, all histidine containing metallopolymers (**MP2 a**, **MP3 a** and **MP4 a**) revealed similar *T*_d_ values which are about 40 °C lower than the *T*_d_ of **MP1 a** which only contains terpyridine ligands. These findings indicate a faster degradation of the histidine ligand compared to terpyridine.

The DSC analysis revealed glass transition temperatures (*T*_g_) in a similar temperature range for all metallopolymers, showing no significant differences between the terpyridine or histidine containing polymers. The found values are slightly below room temperature and very similar to the *T*_g_ of the homopolymer of **PhEtA** (3 °C).^[60]^

Lastly, DMTA was performed using the rectangular shaped metallopolymers **MPXd**. The corresponding DMTA‐curves can be found in **Figures S14** to **S17** (Supporting Information). These measurements showed a constantly crosslinked state of the metallopolymer networks in the investigated temperature range (up to 130 °C). Such a finding can be attributed to a constant network structure of the polymers even at temperatures above the activation of the metal‐ligand complexes, which is comparable to the behavior of vitrimers.^[61]^ For the metallopolymers **MP1d**, **MP2d** and **MP4d** a good retention of the 3D‐structure was observed which is shown by the nice resolution of the obtained DMTA‐curves. Only the sample **MP3d** revealed an increased signal to noise ratio starting at temperatures above 100 °C, which has, however, no influence on the general trend of this measurement. The DMTA measurements revealed the lowest value for the storage modulus G’ at 25 °C for metallopolymer **MP2d** (42 MPa). This confirms the observation made previously, that **MP2** is softer compared to metallopolymer **MP1** (75 MPa) at room temperature. Additionally, it is possible to determine the *T*_g_ at the maximum of the loss modulus G’’ or at the maximum of the loss factor (tan*δ*, **Table** 1).^[62]^ In all DMTA curves G’’ is decreasing at the start of the measurement, and no maximum was found. Therefore, the *T*_g_ values were determined at the maximum of the loss factor. Herein the *T*_g_ values for all metallopolymers were significantly higher than the *T*_g_’s obtained by DSC, which is in good agreement with the literature.^[62]^ While the *T*_g_’s of the metallopolymers **MP1d**, **MP2d** and **MP4d** are in a similar temperature range, the *T*_g_ of metallopolymer **MP3d** is notably higher. Our assumption is that the transition found for sample **MP3d** corresponds to the activation of the trt‐histidine nickel(II) complexes. This might also explain the increasing signal to noise ratio at higher temperatures, due to an increased softening of the metal complex crosslinked sample.

### Thermal Stability of 3D‐Printed Structure

When investigating the self‐healing of metallopolymers in hollow structures it is crucial to ensure the samples retain their 3D‐shape at the intended healing temperature and do not lose their structural integrity upon heating (*i.e*. the material cannot simply only flow to heal the scratch). Therefore, metallopolymers **MP1b**, **MP2b**, **MP3b** and **MP4b** were investigated regarding this issue and were put on a glass carrier and placed in an oven for 24 h at the respective temperatures.

**Figure 2:**
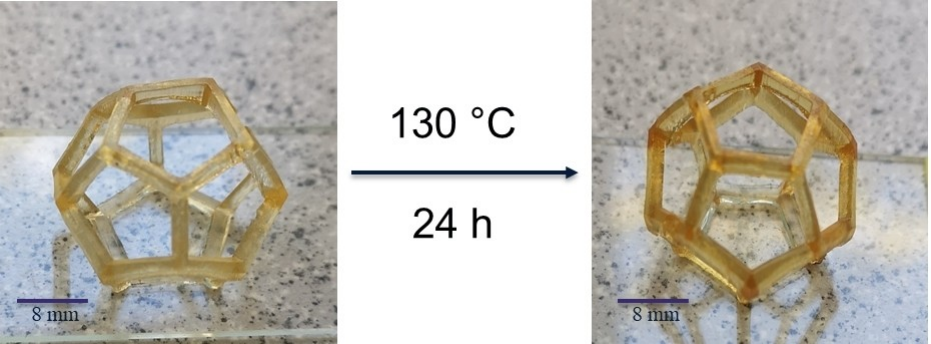
Picture series of the thermal stability of metallopolymer **MP1b** before (left) and after 24 h at 130 °C (right).

The DMTA and TGA measurements discussed in the previous section revealed good thermal stability of the metallopolymers **MP1**, **MP2** and **MP4** up to 130 °C and good thermal stability for metallopolymer **MP3** up to 70 °C. Consequently, the dodecahedrons obtained for the metallopolymers **MP1**, **MP2** and **MP4** were placed in an oven for 24 h at 130 °C. In **Figure** 2 the comparison of metallopolymer **MP1b** before and after 24 h at 130 °C is shown. The respective picture series for the metallopolymers **MP2b**, **MP3b** and **MP4b** can be found in **Figures S18** to **S20** (Supporting Information). After 24 h at 130 °C sample **MP1b** did not show any signs of collapse and retained its 3D‐structure perfectly.

When the metallopolymers **MP2b** and **MP4b** were investigated the same excellent retention of structural integrity after 24 h at 130 °C was observed. Additionally, the samples got darker during the thermal treatment, which is a known phenomenon for 3D‐printed structures and can be related to the decomposition of the photoinitiator during thermal post‐treatment (here BAPO).^[63]^

In case of the metallopolymer **MP3b**, the DMTA curves became slightly noisy at temperatures above 100 °C. Therefore, the investigation of the thermal stability of the dodecahedron was started at 70 °C. Hereafter, the 3D‐structure was nicely retained, and the dodecahedron looked the same as before the thermal treatment (see **Figure S19**). When the temperature was increased to 80 °C, the metallopolymer **MP3b** started to collapse by falling over to the one side. Although the 3D‐structure is not completely lost the healing temperature should not exceed 70 °C to ensure the stability of the dodecahedron.

### Investigation of the Self‐Healing Properties

The aim of this study is the investigation of the self‐healing of 3D‐structures based on supramolecular crosslinking by metal‐ligand complexes. The herein used dodecahedrons (**MPXc**) were obtained by DLP‐based 3D‐printing as described above (see **Figure S1**). The underlaying self‐healing mechanism of metallopolymers is based on dynamic exchange reactions between different metal complexes and is depicted in **Scheme S3** (Supporting Information). This mechanism was already described previously for similar systems.[^49^, ^59^] Moreover, the herein used metal‐ligand combinations were successfully applied previously for the preparation of thin film polymer coatings with the ability to self‐heal introduced damage efficiently.[^13^, ^58^, ^59^]

The materials were glued onto an epoxy block in order to fix them on the surface, enabling a better investigation of the self‐healing behavior. For damaging of the material, a previously described technique, using a microindenter, was applied.^[64]^ This method enabled a damaging of the surface resulting in defined scratches by applying a constant force. Subsequently, the samples were annealed for 24 h at the healing temperature (130 °C for **MP1c**, **MP2c** and **MP4c**, 70 °C for **MP3c**). In previous studies the healing efficiency was determined by 3D‐profile measurements.[^13^, ^65^] However, for the dodecahedrons it was not possible to perform these measurements. Therefore, the healing efficiency was determined by image analysis using ImageJ, which was already utilized in previous studies.[^66^, ^67^]

**Figure 3:**
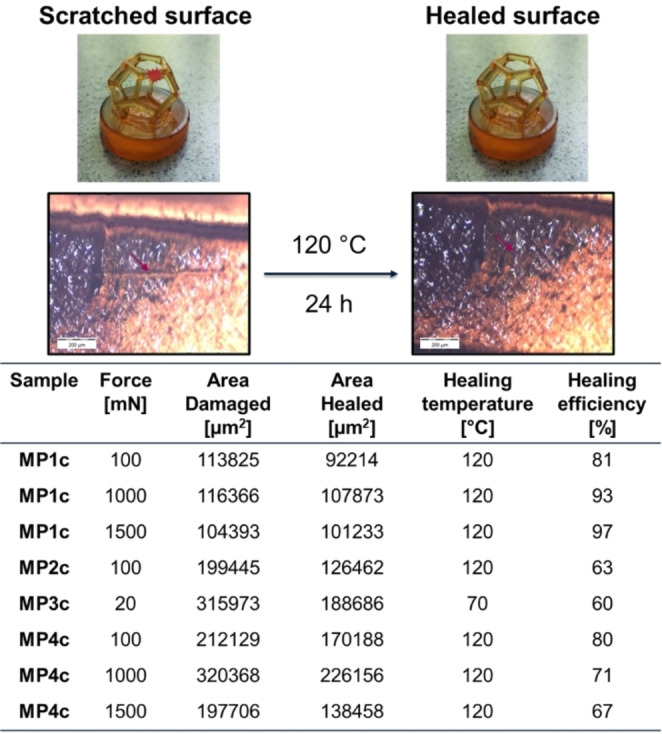
Dodecahedron **MP1c** glued onto an epoxy block before (top left) and after annealing for 24 h at 120 °C (top right); obtained optical images of the metallopolymer **MP1c** scratched with 100 mN of force before (left) and after (right) annealing for 24 h at 120 °C and summarized results of the self‐healing study (bottom).

At first the damages were introduced using a normal force of 100 mN. When the healing efficiency exceeded 70 %, the normal force was increased, and the self‐healing experiment was repeated (up to 1500 mN normal force). An exemplary self‐healing experiment as well as the summarized results of the self‐healing study are depicted in **Figure** 3. The other optical images are displayed in the Supporting Information (**Figures S21** to **S28**).

The investigation of the self‐healing ability revealed that metallopolymer **MP1 c** was the best performing system in this study. This sample is supramolecular crosslinked by terpyridine zinc(II) complexes and reached a healing efficiency of 81 % when the damage was introduced with 100 mN. Increasing the normal force to 1000 mN and 1500 mN respectively, it was possible to increase the self‐healing efficiency even further up to 93 % (1000 mN) and 97 % (1500 mN), respectively.

Metallopolymer **MP2 c** featured only partial self‐healing abilities when a scratch was introduced with 100 mN. Under the same conditions as for **MP1 c** the sample was able to heal 63 % of the scratch area. The substitution of terpyridine with histidine as ligand resulted in a reduced self‐healing efficiency. This is also evident in the optical images since the scratch is clearly visible after thermal treatment (**Figure S23**).

When the surface of metallopolymer **MP3 c** was scratched with 100 mN the surface immediately cracked multiple times (see **Figure S24**). Consequently, it was not possible to introduce a defined scratch. Therefore, the applied normal force was reduced to 20 mN, but still the surface cracked multiple times, and it was not possible to introduce a defined scratch (see **Figure S25**). Nevertheless, self‐healing of the damaged area was attempted and revealed a self‐healing efficiency of 60 %. These findings suggest that supramolecular crosslinking by histidine nickel(II) complexes is not suitable for an efficient self‐healing in 3D‐structure, which is even more evident, since the 3D‐structure of the printed sample is lost during thermal treatment above 80 °C.

Metallopolymer **MP4 c** featured a 1 to 1 mixture of terpyridine‐ and histidine‐zinc(II). This combination of ligands led to high self‐healing efficiencies (80 %), comparable with the sample **MP1 c** when the scratch was introduced with 100 mN of normal force. Damaging the surface with a higher normal force resulted slightly reduced self‐healing behavior. However, in our opinion the healing efficiency could be notably higher (for 1000 mN and 1500 mN).

The obtained images show that the surfaces of the 3D‐printed structures are not perfectly smooth, these irregularities can influence the evaluation *via* ImageJ and, therefore, lead to slight deviations of the determined efficiencies.

Overall, the investigation of self‐healing abilities of metallopolymers obtained by DLP‐based 3D‐printing showed that all samples are able to self‐heal introduced damage while retaining their 3D‐structure. Herein it was found that the samples containing terpyridine complexes enable better self‐healing than samples comprising histidine complexes. This finding is the opposite to the results found in thin films, in which histidine‐based polymers often heal better, *i. e*. at lower temperatures and in shorter times, which was associated with the lower binding efficiency.[^13^, ^49^, ^59^]

Compared to the investigation in thin films, where the polymer backbone consisted only of methacrylates, the herein used 3D‐printed polymeric structures feature a mixture of acrylates and methacrylates in their polymeric backbone. Additionally, the substitution of butyl methacrylate for **PhEtA** greatly reduced the *T*_g_ of the obtained metallopolymers. These variations can result in a change of the structure within the 3D‐printed polymeric material, which might explain the better self‐healing of the terpyridine complex containing polymers.

## Conclusion

Within this work, we demonstrated that DLP‐based 3D‐printing can be applied to obtain metallopolymers in complex structures featuring self‐healing abilities. The herein utilized terpyridine and histidine metal complexes were prepared within the printing mixture before the photopolymerization. The thermal properties of the 3D‐printed networks were studied, and the stability of the 3D‐structure at elevated temperatures was investigated. These metallopolymers were able to self‐heal introduced damages while retaining their 3D‐structure at the respective healing temperatures. Excellent self‐healing efficiencies up to 97 % could be achieved.

In future studies it could be attempted to completely break the struts of the structures to investigate the self‐healing ability on a macroscopic level. Additionally, new metal‐ligand combinations could be researched to further increase the thermal stability of these structures or help tailor the healing temperature.

## Supporting Information

The authors have cited additional references within the Supporting Information.^[68]^

The supporting information contains information about the used chemicals and instruments, the synthesis of the monomers and metallopolymers as well as detailed information about the slice settings used for DLP‐based 3D‐printing. Additionally, the Supporting Information includes optical images of the 3D‐printed structures, TGA‐, DSC‐ and DMTA‐curves of the metallopolymers, as well as picture series of the thermal stability and self‐healing investigations.

## Conflict of Interests

The authors declare no conflict of interest.

## Supporting information

As a service to our authors and readers, this journal provides supporting information supplied by the authors. Such materials are peer reviewed and may be re‐organized for online delivery, but are not copy‐edited or typeset. Technical support issues arising from supporting information (other than missing files) should be addressed to the authors.

Supporting Information
