# Supplementary material for: Self‐Healing Behavior of Metallopolymers in Complex3D‐Structures Obtained by DLP‐Based 3D‐Printing
Source: Chemistry. 2025 Feb 12;31(17):e202404267. doi: 10.1002/chem.202404267 (PMC11924990; doi:10.1002/chem.202404267)
Supplement: Supplementary file 1 — Supporting Information [file CHEM-31-e202404267-s001.pdf]

Chemistry–A European Journal

Supporting Information

Self-Healing Behavior of Metallopolymers in Complex3D-Structures Obtained by DLP-Based 3D-Printing

Michael Klein, Patrick Fesser, Stefan Zechel,* Martin D. Hager,* and Ulrich S. Schubert*

Supporting Information

Self-healing behavior of metallopolymers in complex 3D-structures obtained by DLP-based 3D-printing

Michael Klein,[a,b] Patrick Fesser,[a,b] Stefan Zechel*,[a,b] Martin D. Hager*,[a,b,c] Ulrich S. Schubert*[a,b,c]

[a] M. Klein, P. Fesser, M. D. Hager, S. Zechel, U. S. Schubert
Laboratory of Organic and Macromolecular Chemistry (IOMC)
Friedrich Schiller University Jena
Humboldtstr. 10, Jena 07743, Germany
E-mail: ulrich.schubert@uni-jena.de; martin.hager@uni-jena.de; stefan.zechel@uni-jena.de

[b] M. Klein, P. Fesser, M. D. Hager, S. Zechel, U. S. Schubert
Jena Center for Soft Matter (JCSM)
Friedrich Schiller University Jena
Philosophenweg 7, Jena 07743, Germany

[c] M. D. Hager, U. S. Schubert
Helmholtz-Institute for Polymers in Energy Applications Jena (HIPOLE Jena)
Lessingstr. 12-14, Jena 07443, Germany

Table of contents

Materials and methods S3

Synthesis of the monomers S5

Fmoc-N^{τ}-tritylhistidine butyl amide (2) S5

N^{τ}-Tritylhistidine butyl amide (3) S6

N^{α} -Methacryloyl-N^{τ}-tritylhistidine butyl amide (HistMA) S6

Synthesis of the terpyridine-based monomer S8

6-(2,2:6′,2″-Terpyridin-4′-yloxy)-tetraethylene glycol (5) S8

6-(2,2′:6′,2″-Terpyridin-4′-yloxy)-tetraethylene glycol methacrylate (TpyMA) S9

DLP-based 3D-printing conditions S10

Optical images of the 3D-printed structures S15

Thermogravimetric analysis S17

Differential scanning calorimetry S19

Dynamic mechanical thermo-analysis S21

Thermal stability of 3D-printed structures S24

Self-healing studies S25

References S29

Materials and methods

All chemicals were used as received from TCI (Eschborn, Germany), Thermo Fisher Scientific (Geel, Belgium), Sigma Aldrich (Darmstadt, Germany), Hetcat (Basel, Switzerland), Alfa Aesar (Kandel, Germany) and Acros Organics (Geel, Belgium) if not otherwise stated.

All solvents were dried over molecular sieve under nitrogen atmosphere. Column chromatography was utilizing silica gel 60 from Merck or aluminum oxide (neutral from Molekula) and fraction control was performed using thin-layer chromatography (aluminum sheets coated with silica gel 60 F254 by Merck). Column chromatography was perfomed on a Biotage® Isolera™ One purification system (Biotage, Sweden).

The 3D-models were created in Autodesk Inventor Professional 2022 and exported as a stl-file or downloaded from thingiverse.com as a stl-file. For 3D-printing, the Photon Mono from Anycubic was utilized with self-made modifications for the resin vat. The resin vat with an area of printing of 28 × 28 mm (length, width) was printed out of Nylon filament with an Original Prusa i3 MK3S+ 3D-printer. Under the resin vat a 0.1 mm thick FEP-foil was attached using adhesive tape. As a slicing program for the 3D-printer Photon Workshop V2.1.30x64 was utilized. The curing of the polymers and metallopolymers after 3D-printing was performed in a drying oven from Heraeus Instruments (Hanau, Germany) at 100 °C for 15 h to 22 h.

Thermogravimetric analysis (TGA) was performed under nitrogen atmosphere with a heating rate of 10 K min^{-1} using a Netzsch TG 209 F1 Libra (Selb, Germany). Differential scanning calorimetry (DSC) was measured on a Netzsch 204 F1 Phoenix instrument (Selb, Germany) under a nitrogen atmosphere with a heating rate of 20 K min^{-1} (first and second heating cycle) and 10 K min^{-1} (third heating cycle). The DSC and TGA results were evaluated with the NETZSCH-Proteus-80 software. The glass transition temperature (T_g) from the DSC was determined at the third measurement cycle with a heating rate of 10 K min^{-1}.

Nuclear magnetic resonance (NMR) spectra were measured using a Bruker AC 300 (300 MHz) spectrometer at 298 K. The chemical shift is given in parts per million (ppm on δ-scale) related to deuterated solvent.[1]

All dynamic mechanical thermo-analysis (DMTA) measurements were performed according to a literature procedure.[2] A MCR 301 rheometer from Anton Paar (Graz, Austria) was utilized using the convection oven device CTD 450. The samples were

measured with a solid rectangular fixture setup (SRF12-SN13529, Anton Paar (Graz, Austria)) in dimensions of approximately 24 x 10 mm (length, width) and a thickness of approximately 3.5 to 4.5 mm. The resulting sample gap was set to 12 mm. The software RheoCompassTM V1.24.549-Release 64-bit (Anton Paar, Graz, Austria) was applied for operating the rheometer as well as for analysis. The data was exported as txt-files and evaluated and processed with OriginPro 2019 (OriginLab Corporation, Northampton, MA, USA).

The self-healing scratch tests were performed on an Anton Paar Micro scratch tester MST^3 on a STeP 4 platform. The instrument was equipped with 10 µm Rockwell K-018 indenter and the optical images were taken with the lenses MPlan N 5×/0.10/FN22 and MPlan N 20×/0.40/FN22. The obtained images before and after healing were analyzed using ImageJ.

Synthesis of the monomers

The histidine-based monomer (**HistMA**) was synthesized according to a literature report.[3]

Scheme S1 displays a schematic representation of the synthesis of **HistMA**.

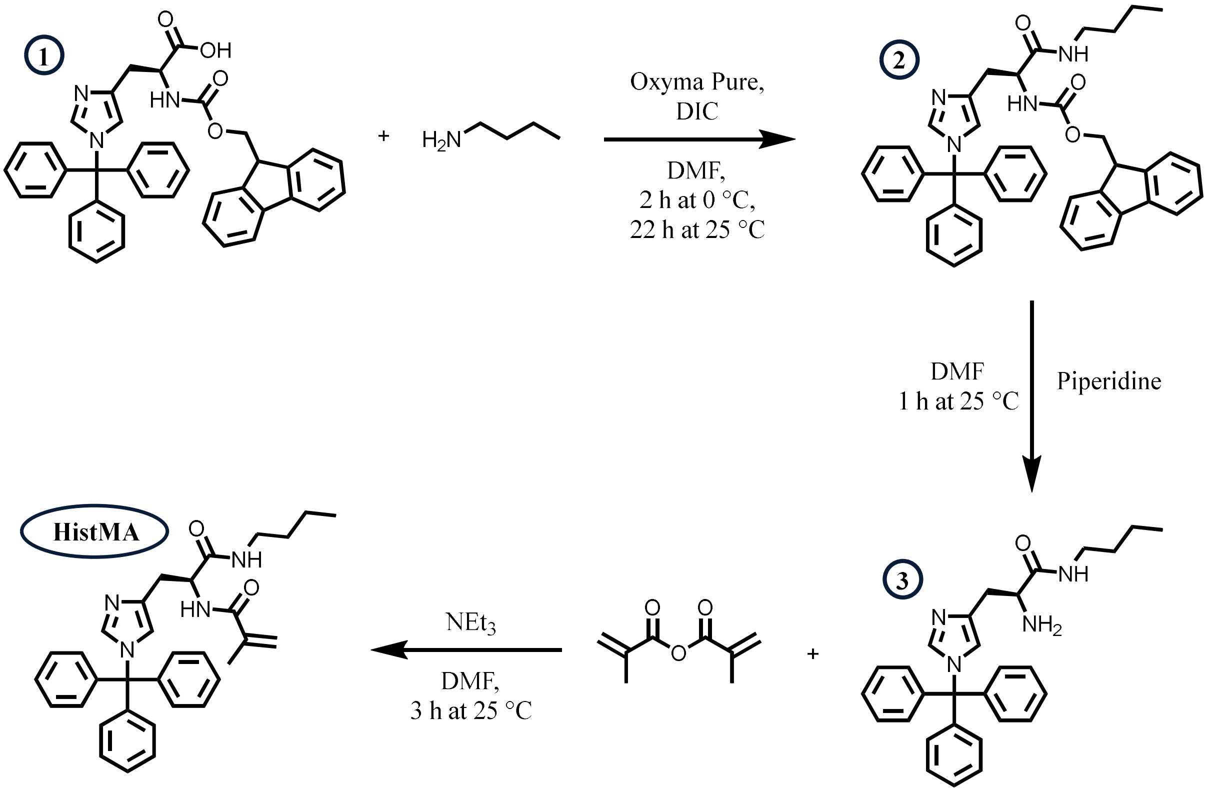

Scheme S1: Schematic representation of the synthesis of the histidine-based monomer **HistMA**. DIC = *N*,*N*'-Diisopropyl carbodiimide. DMF = *N*,*N*'-dimethyl formamide.

Fmoc-*N*^{τ}-tritylhistidine butyl amide (2)

The synthesis was performed under nitrogen atmosphere according to a literature report.[3] Fmoc-His-(Trt)-OH **1** (1.0 eq, 5.00 g, 8.07 mmol) was dissolved in 20 mL dry dimethylformamide (DMF) and cooled to 0 °C. Successively butylamine (1.0 eq, 0.59 g, 8.07 mmol) and Oxyma Pure (1.0 eq, 1.15 g, 8.07 mmol) were added and the solution stirred for 15 min. Afterwards *N,N*^{τ}-diisopropylcarbodiimide (DIC, 1.0 eq, 1.02 g, 8.07 mmol) was added and the reaction mixture was stirred for 2 h at 0 °C and 22 h at ambident temperature. Subsequently, the solvent DMF was evaporated *in vacuo* and the residue was redissolved in 200 mL chloroform, washed with brine (3 × 200 mL) and subsequently dried over sodium sulfate. Silica gel chromatography

(CH_2Cl_2/MeOH 95/5 (R_f = 0.6)) was performed, and the obtained yellow solid was used without further purification (5.909 g).

N^τ-Tritylhistidine butyl amide (3)

The synthesis was performed according to a literature procedure.[3]
2 (1.0 eq, 5.909 g, 8.76 mmol) was dissolved in 40 mL dry DMF. Piperidine (4.0 eq, 2.98 g, 35.02 mmol) was added and the solution turned orange. After stirring for 60 min at ambient temperature (full consumption of **2** was confirmed by thin layer chromatography (TLC)) the reaction mixture was concentrated *in vacuo*. The crude product was purified by automated silica gel chromatography (Isolera; CH_2Cl_2/MeOH 95/5 (R_f = 0.8)) yielding **3** as a yellow solid (2.49 g, 68% over two steps).

^{1}H NMR (300 MHz, $CDCl_3$, δ): 0.89 (t, J = 4.4 Hz, 3H, -CH_3), 1.27-1.37 (m, 2H, -CH_2-CH_2-CH_3), 1.40-1.47 (m, 2H, -CH_2-CH_2-CH_3), 2.74-3.06 (m, 2H, -CH_2-), 3.11-3.25 (m, 2H, -CONH-CH_2-), 3.63 (q, J = 2.6 Hz, 1H, -C*H*-), 6.64 (d, J = 0.8 Hz, 1H *H*-Im_2), 7.08-7.12 (m, 6H, *H*-aryl), 7.28-7.34 (m, 9H, *H*-aryl), 7.36 (d, J = 0.8 Hz, 1H, *H*-Im_5), 7.48 (t, J = 3.3 Hz, 1H, -N*H*-) ppm.

N^α -Methacryloyl-N^τ-tritylhistidine butyl amide (HistMA)

The synthesis was performed under nitrogen atmosphere according to a literature report.[3]
3 (1.0 eq, 3.922 g, 8.67 mmol) was dissolved in 90 mL dry dichloromethane. Triethylamine (2.0 eq, 1.75 g, 17.33 mmol) was added and the mixture stirred for 10 min. Hereafter, methacrylic anhydride (2.0 eq, 2.67 g, 17.33 mmol) was added dropwise. After 3 h full conversion of **3** was confirmed by TLC. The solvent was evaporated and the solid residual was redissolved in 100 mL dichloromethane. Subsequently, the organic solution was washed with a saturated solution of sodium hydrogen carbonate (2 × 100 mL) and brine (100 mL) with subsequent drying over sodium sulfate. For purification silica gel chromatography (EtOAc (R_f = 0.4)) was performed yielding **HistMA** as a white solid (3.14 g, 69%).

^{1}H NMR (300 MHz, $CDCl_3$, δ): 0.88 (t, J = 7.2 Hz, 3H, -CH_3), 1.22-1.49 (m, 4H, -CH_2-CH_2-CH_3), 1.98 (s, 3H, -CH_3), 2.04 (s, 1H, -N*H*-), 2.88-3.25 (m, 4H, -CH_2-, -CONH-

CH_2-), 4.69 (q, *J* = Hz, 1H, -C*H*-), 5.37 (s, 1H, =CH_2), 5.82 (s, 1H, =CH_2), 6.68 (s, 1H, *H*-Im$_2$), 7.04-7.14 (m, 6H, *H*-aryl), 7.28-7.36 (m, 9H, *H*-aryl), 7.40 (s, 1H, *H*-Im$_5$), 7.89 (d, *J* = 6.1 Hz, 1H, -N*H*-) ppm.

Synthesis of the terpyridine-based monomer

The terpyridine-based monomer (**TpyMA**) was synthesized according to a literature report.[4]

The synthetic pathway is depicted in **Scheme *S2***.

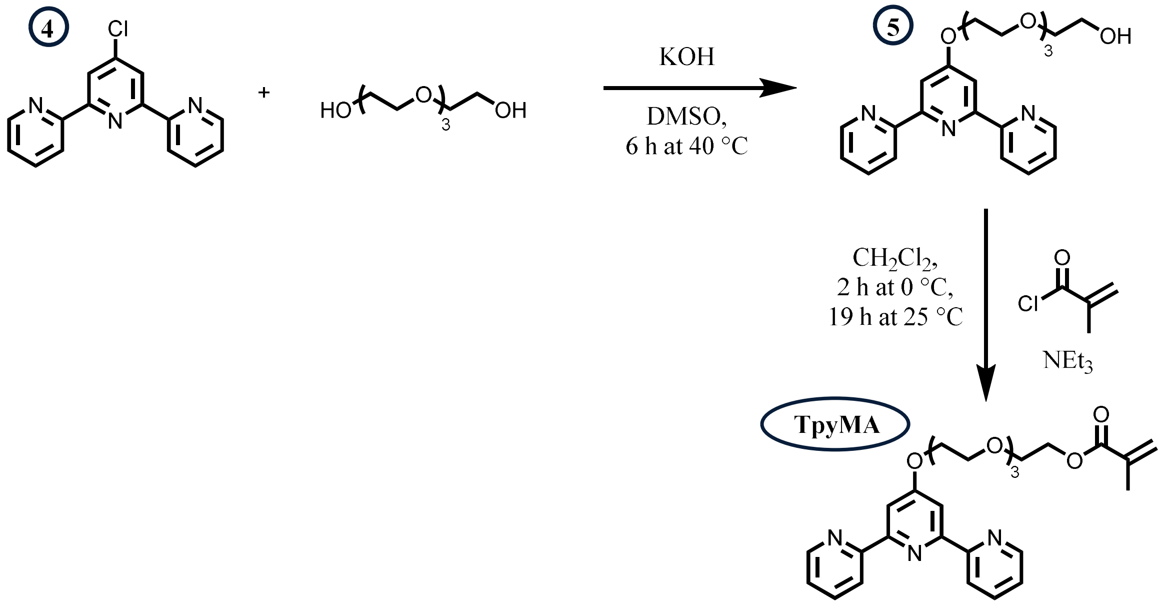

Scheme S2: Schematic representation of the synthesis of the terpyridine-based monomer **TpyMA**. DMSO = dimethyl sulfoxide.

6-(2,2:6′,2″-Terpyridin-4′-yloxy)-tetraethylene glycol (5)

The synthesis was performed according to a literature procedure.[4]

Potassium hydroxide (5.0 eq, 3.20 g, 57.03 mmol) was suspended in 75 mL dimethyl sulfoxide at 40 °C. After 15 min, tetraethylene glycol (10.0 eq, 21.77 g, 112.10 mmol) was added and the mixture stirred for 40 min. Hereafter, 4'-chloro-2,2':6',2''-terpyridine **4** (1.0 eq, 3.04 g, 11.21 mmol) was added. After stirring for 6 h at 40 °C, the solution was poured onto 1.6 L of distilled water. After three days, the precipitate was removed by filtration and the aqueous phase was extracted with dichloromethane (3 × 200 mL). The combined organic fractions were washed with a saturated solution of sodium hydrogen carbonate (200 mL) and brine (200 mL) and subsequently dried over sodium sulfate. Concentration *in vacuo* yielded **5** as a colorless, viscous oil (4.54 g, 95%).

^1H NMR (300 MHz, $CDCl_3$, *δ*): 3.57-3.79 (m, 12H, -C*H*$_2$-O-), 3.93 (t, *J* = 4.65 Hz, 2H, -C*H*$_2$-OH), 4.41 (t, *J* = 4.65 Hz, 2H, -C*H*$_2$-O-), 7.33 (m, 2H, *H*-aryl), 7.85 (t, *J* = 7.8 Hz, 2H, *H*-aryl), 8.04 (s, 2H, *H*-aryl), 8.62 (d, *J* = 7.8 Hz, 2H, *H*-aryl), 8.68 (m, 2H, *H*-aryl) ppm.

6-(2,2′:6′,2″-Terpyridin-4′-yloxy)-tetraethylene glycol methacrylate (TpyMA)

The synthesis was performed under nitrogen atmosphere according to a literature report.[4]

5 (1.0 eq, 4.54 g, 10.66 mmol) was dissolved in 220 mL dry dichloromethane and triethylamine (3.0 eq, 3.29 g, 32.46 mmol) was added. After stirring for 30 min, the solution was cooled to 0 °C and methacrylic chloride (2.0 eq, 2.28 g, 21.81 mmol) was added dropwise at 0 °C. The reaction mixture was stirred for 2 h at 0 °C and subsequently 19 h at ambient temperature. The solution was concentrated *in vacuo* and the viscous residual was redissolved in 200 mL dichloromethane. The organic solution was washed with a saturated solution of sodium hydrogen carbonate (2 × 200 mL) and brine (200 mL). Hereafter, the organic phase was dried over sodium sulfate and concentrated *in vacuo*. Silica gel chromatography (CH_2Cl_2/MeOH 9/1 (R_f = 0.3)) yielded **TpyMA2** as a yellow oil (4.539 g, 86%).

^1H NMR (300 MHz, $CDCl_3$, *δ*): 1.93 (s, 3H, -C*H*$_3$), 3.62-3.78 (m, 10 H, -C*H*$_2$-O-), 3.93 (t, *J* = 4.6 Hz, 2H, -C*H*$_2$-O-), 4.29 (t, *J* = 4.8 Hz, -C*H*$_2$-O-), 4.40 (t, *J* = 4.6 Hz, 2H, -C*H*$_2$-O-), 5.55 (s, 1H, =C*H*$_2$), 6.11 (s, 1H, =C*H*$_2$), 7.33 (p, *J* = 6.1 Hz, *H*-aryl), 7.85 (t, *J* = 7.5 Hz, 2H, *H*-aryl), 8.03 (s, 2H, *H*-aryl), 8.61 (dd, *J* = 8 Hz, 2H, *H*-aryl), 8.69 (td, *J* = 4.8 Hz, 2H, *H*-aryl) ppm.

DLP-based 3D-printing conditions

All samples were printed according to a literature procedure.[4]

All 3D-prints were carried out on a Photon Mono 3D-printer from Anycubic. The used slice settings are summarized in **Table S1**. The monomers and photoinitiator phenyl-*bis*(2,4,6-trimethylbenzoyl) phosphine oxide (BAPO) were mixed in the absence of light until the photoinitiator was fully dissolved. After printing, the obtained structures were washed with acetone and, subsequently, cured at 100 °C for 15 h to 22 h in a drying oven (**MP1a** to **MP1d**, **MP2a** to **MP2d** and **MP4a** to **MP4d**). The metallopolymers **MP3a** to **MP3d** were cured in an UV-cube for 30 minutes.

Table S1: Slice settings utilized for 3D-printing with the Photon Mono 3D-printer from Anycubic.

**Settings**	
**Layer thickness [mm]**	0.050
**Normal exposure time [s]**	20.000
**Off time [s]**	1.000 or 2.000[a]
**Bottom exposure time [s]**	25.000
**Bottom layers**	6
**Z-lift distance [mm]**	6.00
**Z-lift speed [mm s^{-1}]**	2.00[a] or 4.00
**Z-retract speed [mm s^{-1}]**	6.00
**Anti-alias**	1

a) Printing conditions for **MP3a**, **MP3b** and **MP3c**.

The 3D-model of the dodecahedron printed in this work was downloaded from thingiverse.com as a stl-file. The rectangular shape was created using Autodesk Inventor Professional 2022 and exported *via* a stl-file. Hereafter, the files were imported into the photon workshop x86 provided by Anycubic. The 3D-models utilized in this work are depicted in **Figure S1**.

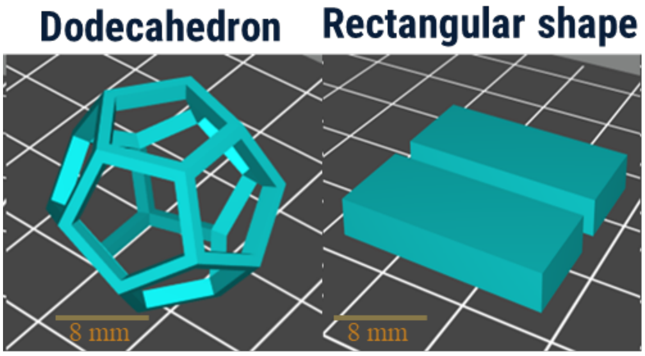

Figure S1: Depiction of the 3D-models utilized for the 3D-printing.

In **Table S2**, the conditions for the 3D-printing of the metallopolymers **MP1a** to **MP4d** are summarized. A BAPO to 2-phenoxyethyl acrylate (PhEtA) ratio of 1:1100 was utilized. The stabilizer (MEHQ) added by the manufacturers TCI was not removed (not specified for PhEtA).

For the preparation of the metallopolymers, the ligand monomers were dissolved in PhEtA. The metal salt (zinc(II) trifluoromethanesulfonate ($Zn(OTf)_2$) or nickel(II) chloride ($NiCl_2$)) was dissolved in 10 mL methanol and added to the monomer mixture. After stirring for 10 min at ambient temperature the methanol was evaporated *in vacuo*. Subsequently, the mixture was added to BAPO and stirred under the exclusion of light until the photoinitiator was fully dissolved. Hereafter the mixture was poured into the resin vat and 3D-printed.

Table S2: Summarized conditions for the 3D-printing of the metallopolymers **MP1a** to **MP4d**.

Print	Model	Monomers		Metal salt		BAPO
**MP1a**	**Dodecahedron**	PhEtA<br>TpyMA	2.554 g (13.29 mmol)<br>0.654 g (1.33 mmol)	$Zn(OTf)_2$	0.242 g (0.66 mmol)	5.0 mg (0.012 mmol)
**MP1b**	**Dodecahedron**	PhEtA<br>TpyMA	2.553g (13.28 mmol)<br>0.650 g (0.133 mmol)	$Zn(OTf)_2$	0.241 g (0.66 mmol)	5.1 mg (0.012 mmol)
**MP1c**	**Dodecahedron**	PhEtA<br>TpyMA	2.557 g (13.30 mmol)<br>0.652 g (0.133 mmol)	$Zn(OTf)_2$	0.240 g (0.66 mmol)	5.0 mg (0.012 mmol)
**MP1d**	**Rectangular shape**	PhEtA<br>TpyMA	2.550 g (13.28 mmol)<br>0.650 g (1.33 mmol)	$Zn(OTf)_2$	0.239 g (0.66 mmol)	5.0 mg (0.012 mmol)
**MP2a**	**Dodecahedron**	PhEtA<br>HistMA	2.557 g (13.30 mmol)<br>0.691 g (1.33 mmol)	$Zn(OTf)_2$	0.240 g (0.66 mmol)	5.0 mg (0.012 mmol)
**MP2b**	**Dodecahedron**	PhEtA<br>HistMA	2.558 g (13.31 mmol)<br>0.694 g (1.33 mmol)	$Zn(OTf)_2$	0.241 g (0.66 mmol)	5.0 mg (0.012 mmol)
**MP2c**	**Dodecahedron**	PhEtA<br>HistMA	2.553 g (13.28 mmol)<br>0.693 g (1.33 mmol)	$Zn(OTf)_2$	0.241 g (0.66 mmol)	5.0 mg (0.012 mmol)
**MP2d**	**Rectangular shape**	PhEtA<br>HistMA	2.552 g (13.28 mmol)<br>0.692 g (1.33 mmol)	$Zn(OTf)_2$	0.239 g (0.66 mmol)	5.0 mg (0.012 mmol)
**MP3a**	**Dodecahedron**	PhEtA<br>HistMA	2.551 g (13.28 mmol)<br>1.037 g (1.99 mmol)	$NiCl_2 \times 6 H_2O$	0.158 g (0.99 mmol)	5.1 mg (0.012 mmol)

Print	Model	Monomers		Metal salt		BAPO
**MP3b**	**Dodecahedron**	PhEtA<br>HistMA	2.557 g (13.30 mmol)<br>1.035 g (1.99 mmol)	$NiCl_2$ × 6 H_2O	0.158 g (0.66 mmol)	5.1 mg (0.012 mmol)
**MP3c**	**Dodecahedron**	PhEtA<br>HistMA	2.557 g (13.30 mmol)<br>1.037 g (1.99 mmol)	$NiCl_2$ × 6 H_2O	0.157 g (0.66 mmol)	5.2 mg (0.012 mmol)
**MP3d**	**Rectangular shape**	PhEtA<br>HistMA	2.552 g (13.28 mmol)<br>1.034 g (1.99 mmol)	$NiCl_2$ × 6 H_2O	0.156 g (0.66 mmol)	5.0 mg (0.012 mmol)
**MP4a**	**Dodecahedron**	PhEtA<br>TpyMA<br>HistMA	2.559 g (13.31 mmol)<br>0.325 g (0.66 mmol)<br>0.345 g (0.66 mmol)	$Zn(OTf)_2$	0.241 g (0.66 mmol)	5.1 mg (0.012 mmol)
**MP4b**	**Dodecahedron**	PhEtA<br>TpyMA<br>HistMA	2.554 g (13.29 mmol)<br>0.326 g (0.66 mmol)<br>0.347 g (0.66 mmol)	$Zn(OTf)_2$	0.242 g (0.66 mmol)	5.0 mg (0.012 mmol)
**MP4c**	**Dodecahedron**	PhEtA<br>TpyMA<br>HistMA	5.116 g (26.56 mmol)<br>0.651 g (1.32 mmol)<br>0.688 g (1.32 mmol)	$Zn(OTf)_2$	0.484 g (1.33 mmol)	10.4 mg (0.025 mmol)
**MP4d**	**Rectangular shape**	PhEtA<br>TpyMA<br>HistMA	2.558 g (13.31 mmol)<br>0.326 g (0.66 mmol)<br>0.345 g (0.66 mmol)	$Zn(OTf)_2$	0.241 g (0.66 mmol)	5.0 mg (0.012 mmol)

Elemental analysis:

	MP1a		MP2a		MP3a		MP4a	
	Theoretical	Measured	Theoretical	Measured	Theoretical	Measured	Theoretical	Measured
**C [%]**	65.01	61.24	65.74	63.39	68.03	68.28	64.49	62.62
**H [%]**	5.97	5.67	6.02	5.75	6.23	6.27	6.47	5.83
**N [%]**	1.65	2.01	2.14	2.85	2.98	2.68	1.87	2.27
**S [%]**	1.26	1.50	1.22	1.44	-	-	2.17	1.50
**Cl [%]**	-	-	-	-	1.26	1.37	-	-

Optical images of the 3D-printed structures

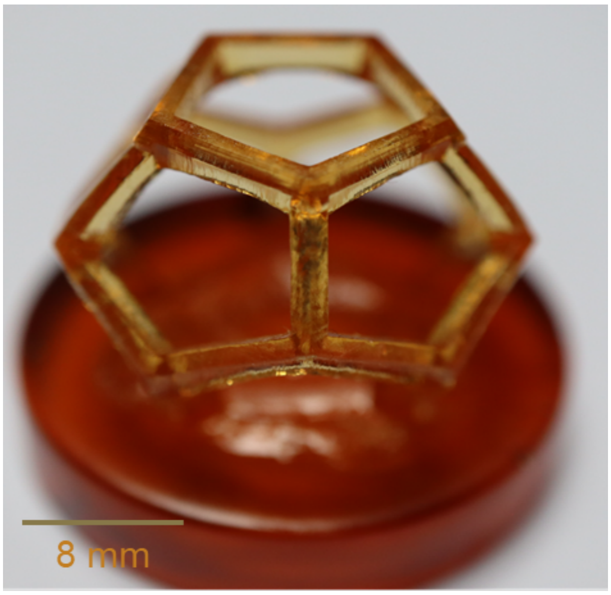

Figure S2: Optical image of one strut intersection of metallopolymer **MP1c**.

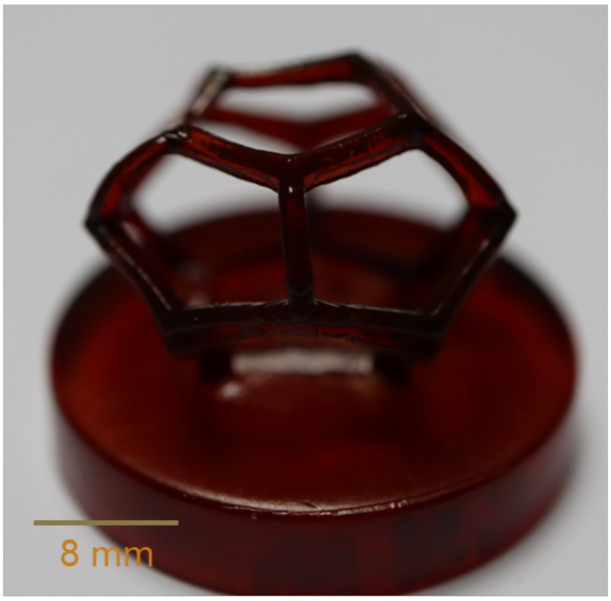

Figure S3: Optical image of one strut intersection of metallopolymer **MP2c**.

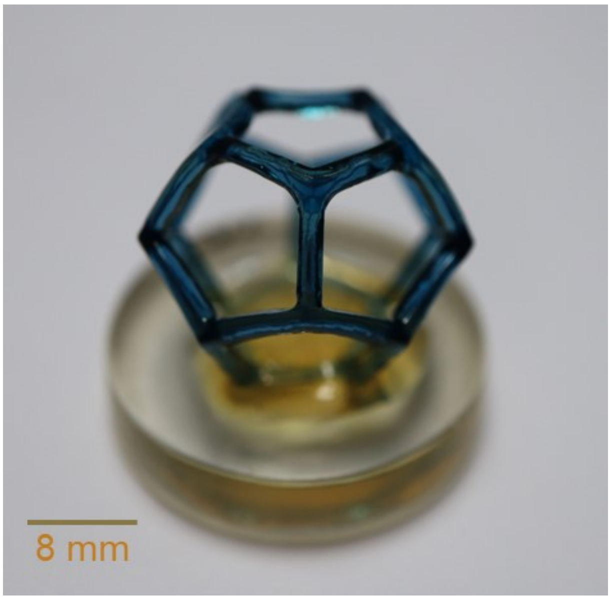

Figure S4: Optical image of one strut intersection of metallopolymer **MP3c**.

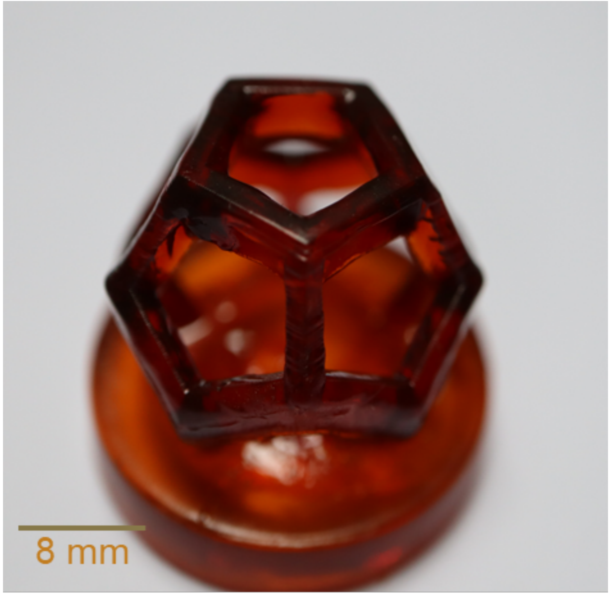

Figure S5: Optical image of one strut intersection of metallopolymer **MP4c**.

Thermogravimetric analysis

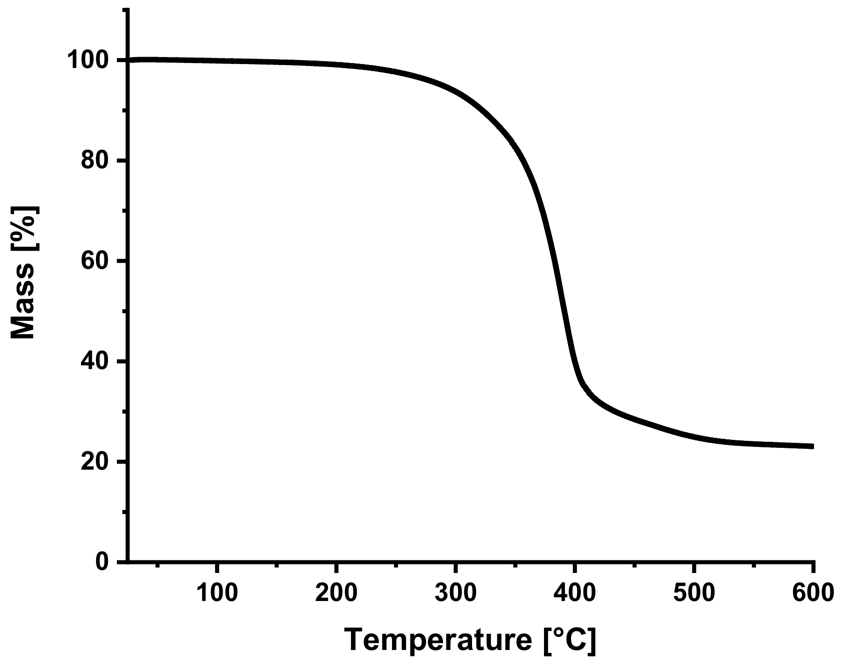

Figure S6: TGA-curve of the metallopolymer **MP1a** (heating rate = 10 K min^{-1}).

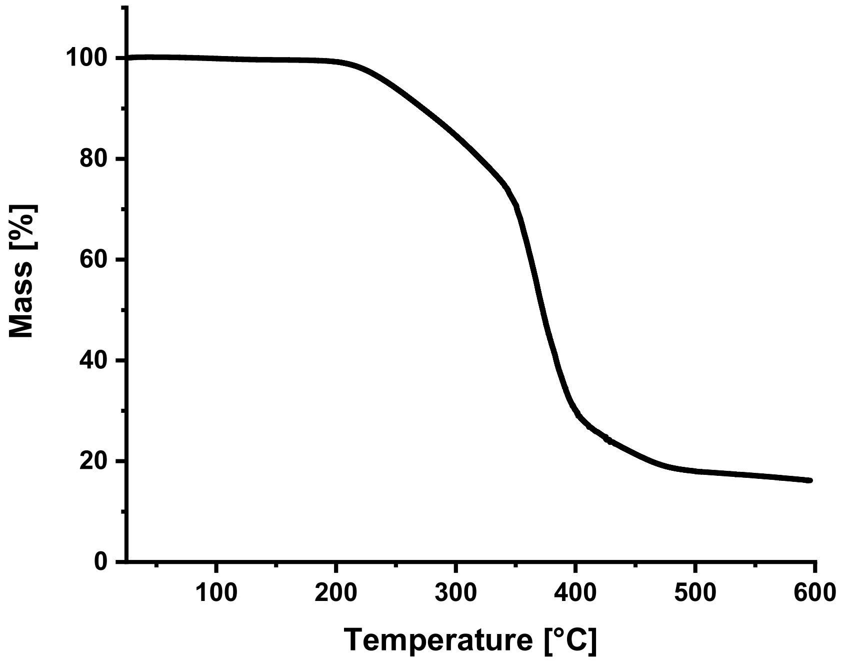

Figure S7: TGA-curve of the metallopolymer **MP2a** (heating rate = 10 K min^{-1}).

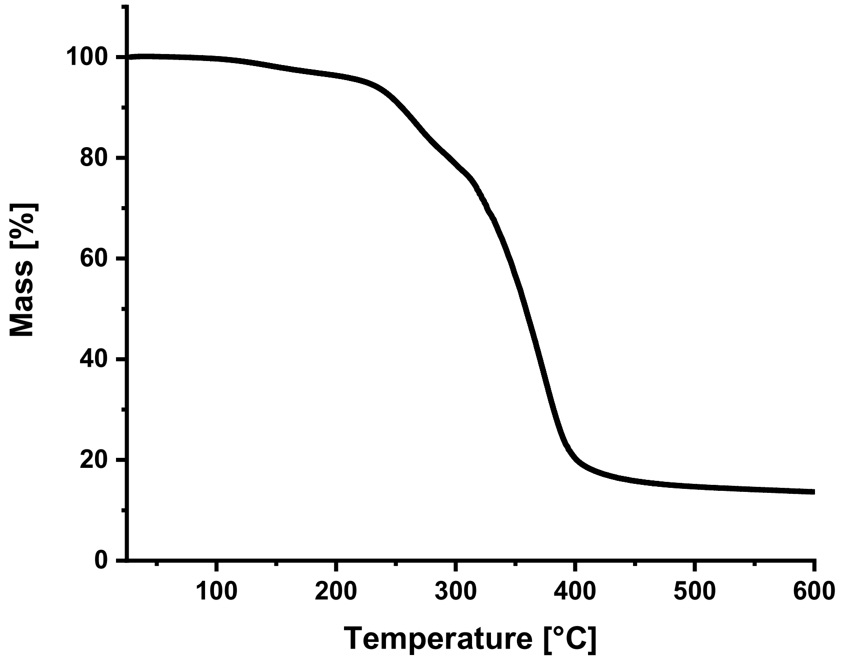

Figure S8: TGA-curve of the metallopolymer **MP3a** (heating rate = 10 K min^{-1}).

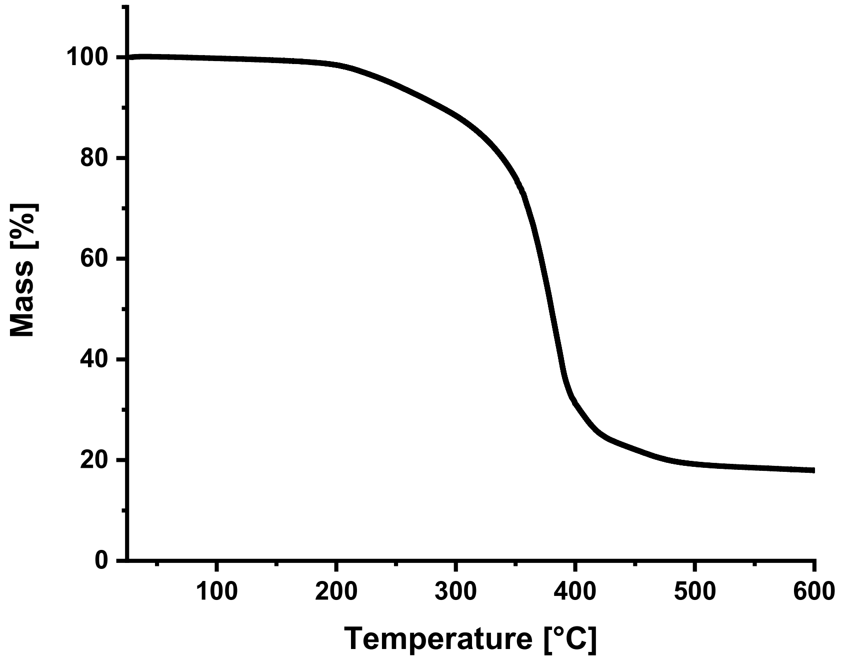

Figure S9: TGA-curve of the metallopolymer **MP4a** (heating rate = 10 K min^{-1}).

Differential scanning calorimetry

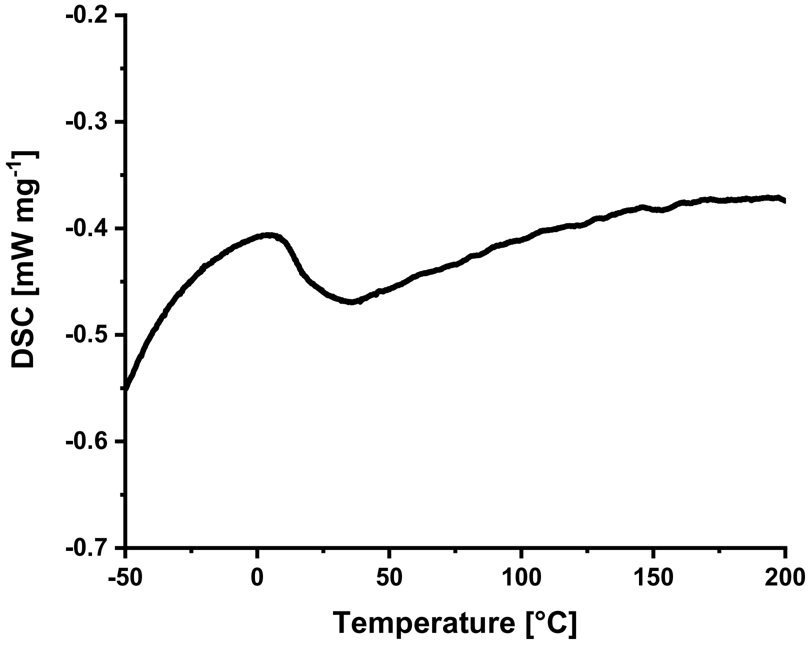

Figure S10: DSC-curve of the third heating cycle (heating rate = 10 K min^{-1}) of the metallopolymer **MP1a**.

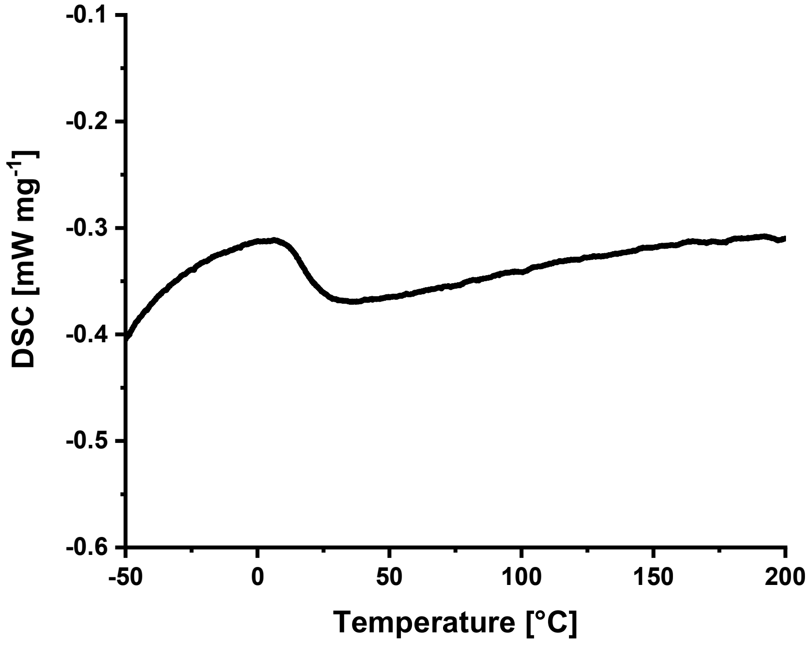

Figure S11: DSC-curve of the third heating cycle (heating rate = 10 K min^{-1}) of the metallopolymer **MP2a**.

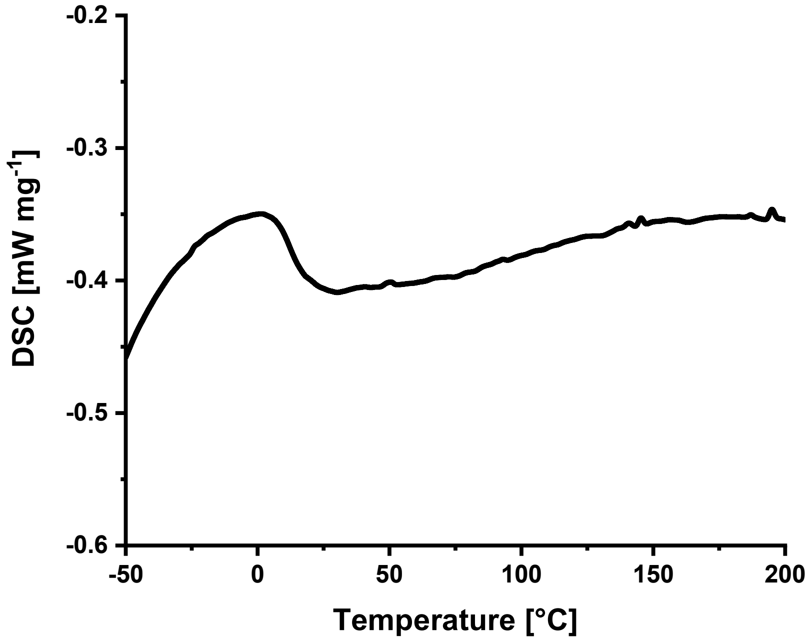

Figure S12: DSC-curve of the third heating cycle (heating rate = 10 K min^{-1}) of the metallopolymer **MP3a**.

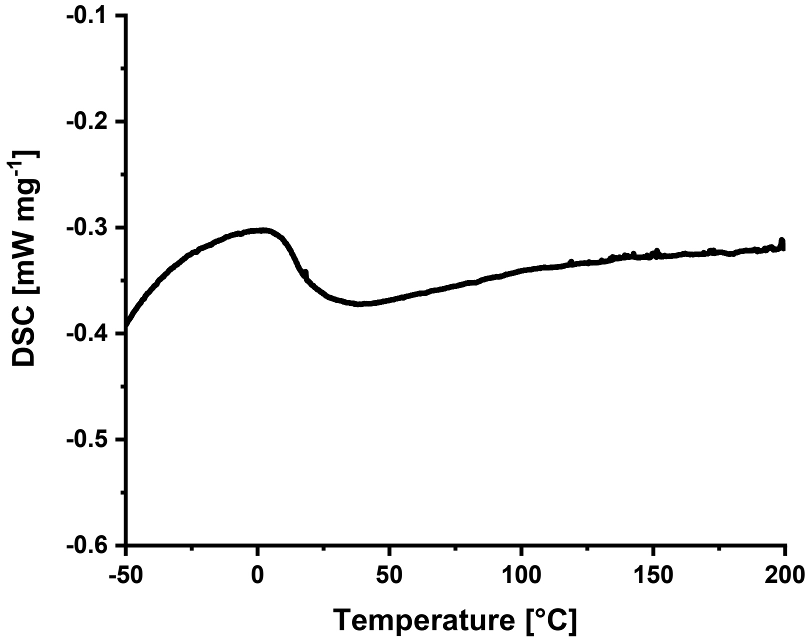

Figure S13: DSC-curve of the third heating cycle (heating rate = 10 K min^{-1}) of the metallopolymer **MP4a**.

Dynamic mechanical thermo-analysis

After fixing the sample, with dimensions of approximately 24 x 10 mm (length, width) and a thickness of approximately 3.5 to 4.5 mm, into the rheometer the resulting sample gap was set to 12 mm. The samples were set to 25 °C and heated up to 130 °C with a heating rate of 2 K min^{-1}, a frequency of 1 Hz and 0.01% sheer strain.

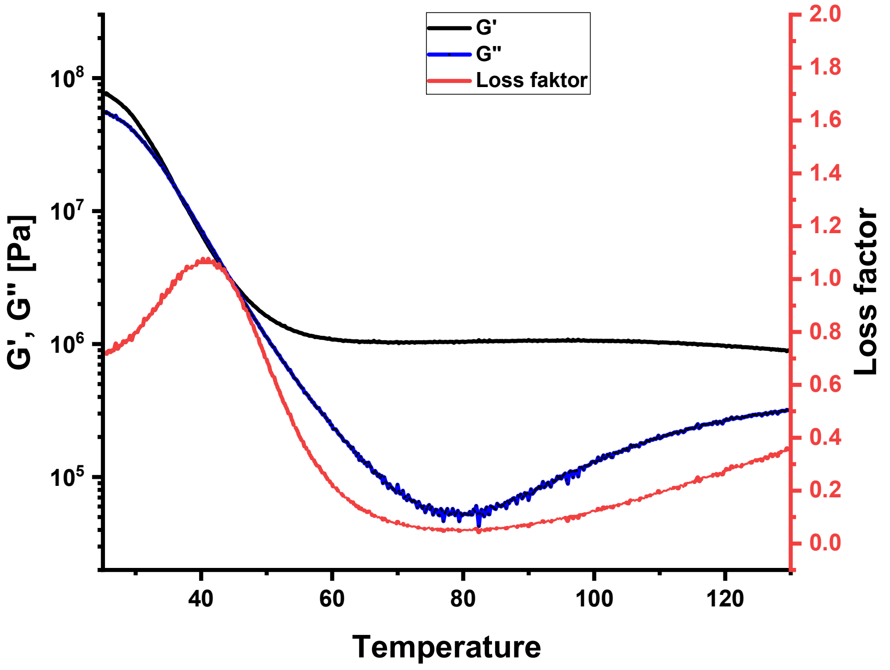

Figure S14: DMTA-curve of the metallopolymer **MP1d**.

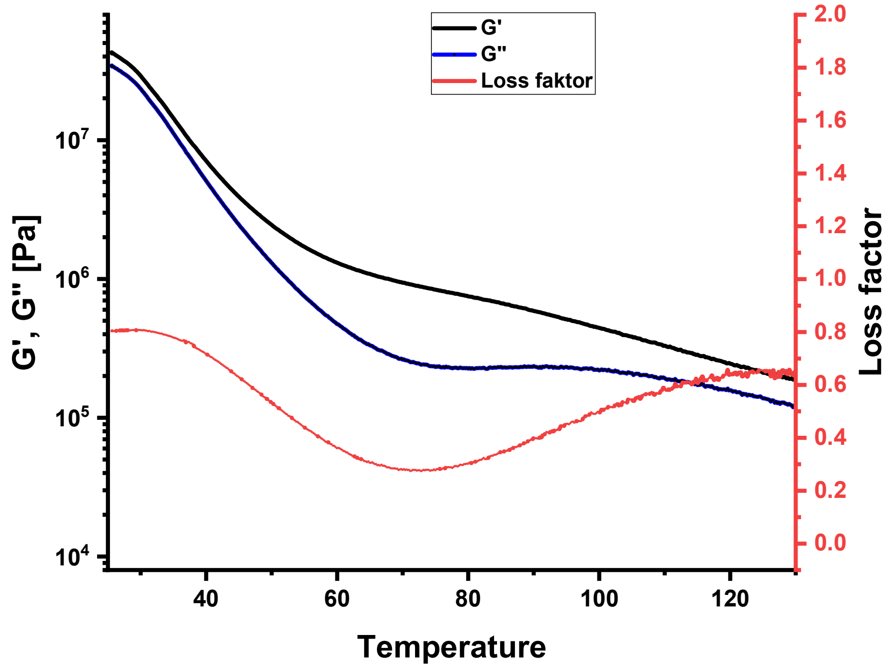

Figure S 15: DMTA-curve of the metallopolymer **MP2d**.

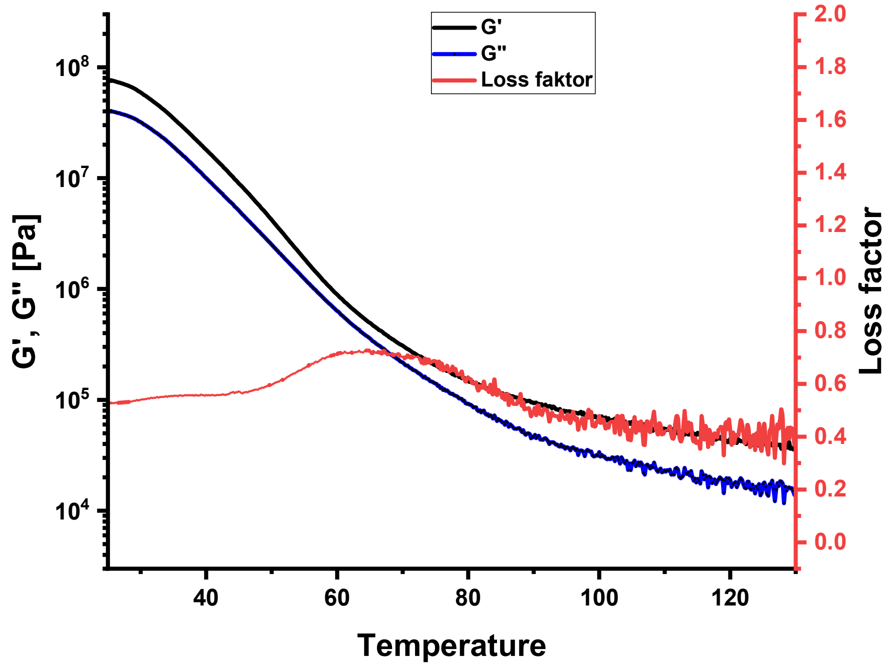

Figure S16: DMTA-curve of the metallopolymer **MP3d**.

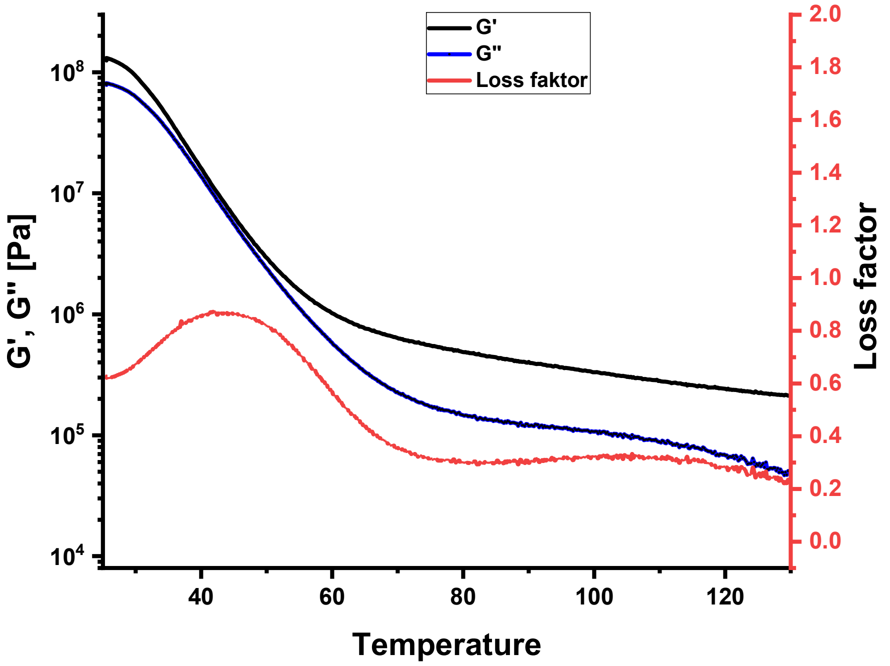

Figure S17: DMTA-curve of the metallopolymer **MP4d**.

Thermal stability of 3D-printed structures

The dodecahedrons obtained by DLP-based 3D-printing of the metallopolymers **MPXb** were placed on a glass carrier and placed in an oven for 24 h at the respective temperatures.

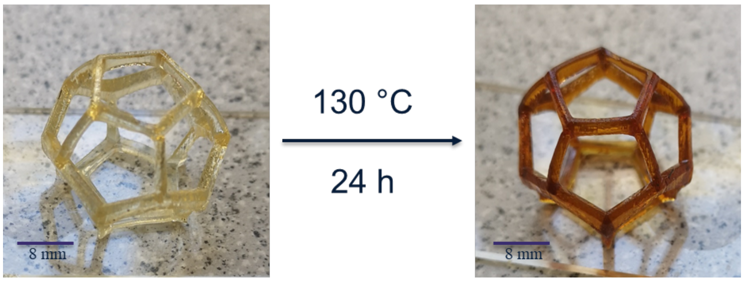

Figure S18: Picture series of the thermal stability of metallopolymer **MP2b** before (left) and after 24 h at 130 °C (right).

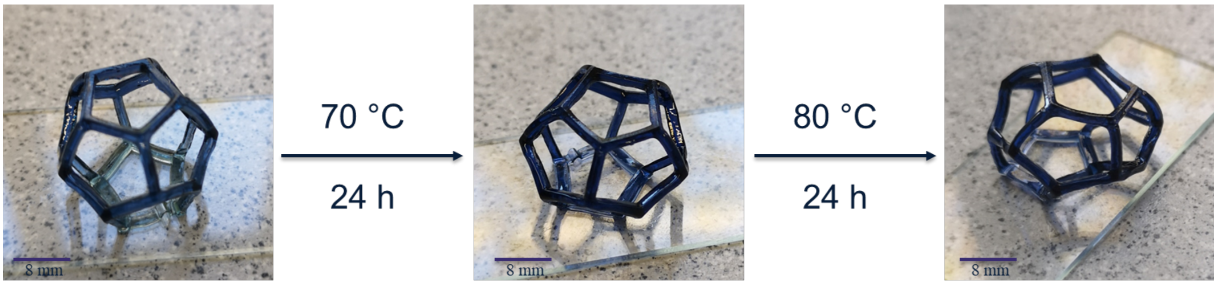

Figure S19: Picture series of the thermal stability of metallopolymer **MP3b** before (left), after 24 h at 70 °C (middle) and loss of structural integrity after 24 h at 80 °C (left).

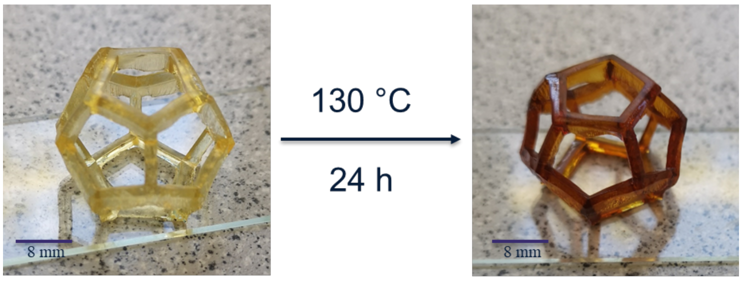

Figure S20: Picture series of the thermal stability of metallopolymer **MP4b** before (left) and after 24 h at 130 °C (right).

Self-healing studies

The scratch healing tests were performed on a micro scratch tester (MST^3, Anton Paar). The instrument is fitted with an indenter, which can be equipped with different indenter tips. Additionally, the MST^3 has a microscope nosepiece with different lenses to image the scratches afterwards (5×, 20×). The samples **MP1c**, **MP2c**, **MP3c**, and **MP4c** were glued onto an epoxy block (Epoxy Resin L and Hardener CL from R&G Faserverbundstoffe GmbH, Waldenburg, Germany) and placed in a (rotatable) sample holder, which has an inlet for an LED light under the transparent sample to additionally illuminate the polymer from below. The scratches were performed with a 10 µm Rockwell indenter, using a normal force of either 20 mN, 100 mN, 1000 mN or 1500 mN, and 1 passage on a length of 1000 µm and a scratch speed of 30000 µm min^{-1}. Subsequently, the scratch was imaged by the microscope. Hereafter, the samples were placed in a pre-heated oven at 70 °C (for **MP3c**) or 120 °C (for **MP1c**, **MP2c** and **MP4c**) for 24 h and imaged by the microscope again.

The obtained images before and after healing were analyzed using ImageJ. The edge length of the scratches of 1000 µm was used as a reference. As the contrast of the obtained images was not sufficient for automatic detection, the areas were analyzed manually. They were outlined by hand and the area was determined using the software ImageJ. This was carried out for all samples. The area of the healed surface in relation to the scratched surface was herein used to determine the healing efficiency according to equation (1).

$$H_{eff} = \frac{A_{healed}}{A_{damaged}} \text{ x } 100 \qquad (1)$$

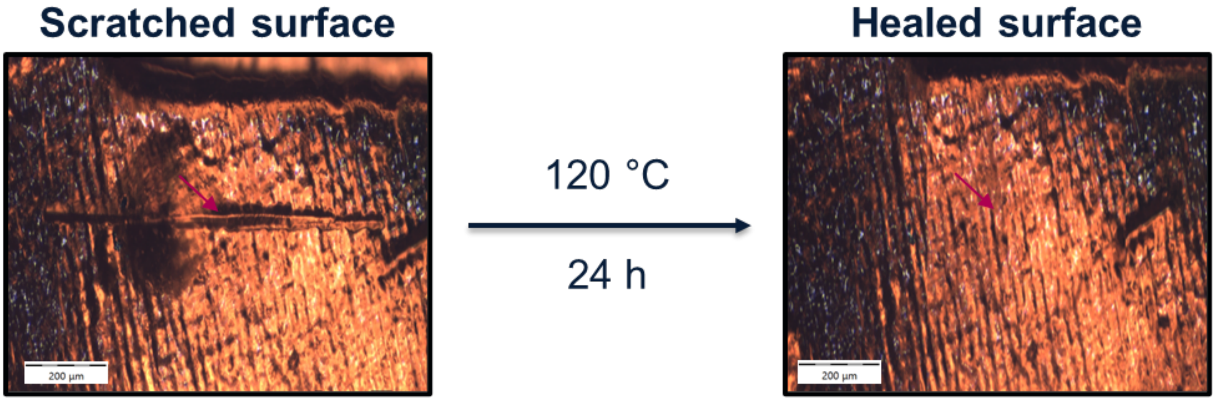

Figure S21: Optical images of the scratch on the surface introduced with a normal force of 1000 mN (left) and the healed scratch after 24 h at 120 °C (right) for metallopolymer **MP1c**.

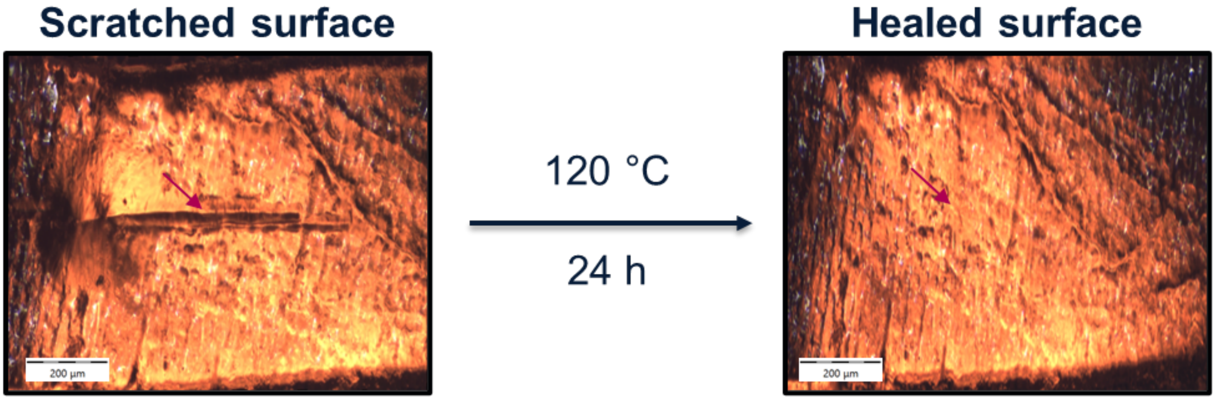

Figure S22: Optical images of the scratch on the surface introduced with a normal force of 1500 mN (left) and the healed scratch after 24 h at 120 °C (right) for metallopolymer **MP1c**.

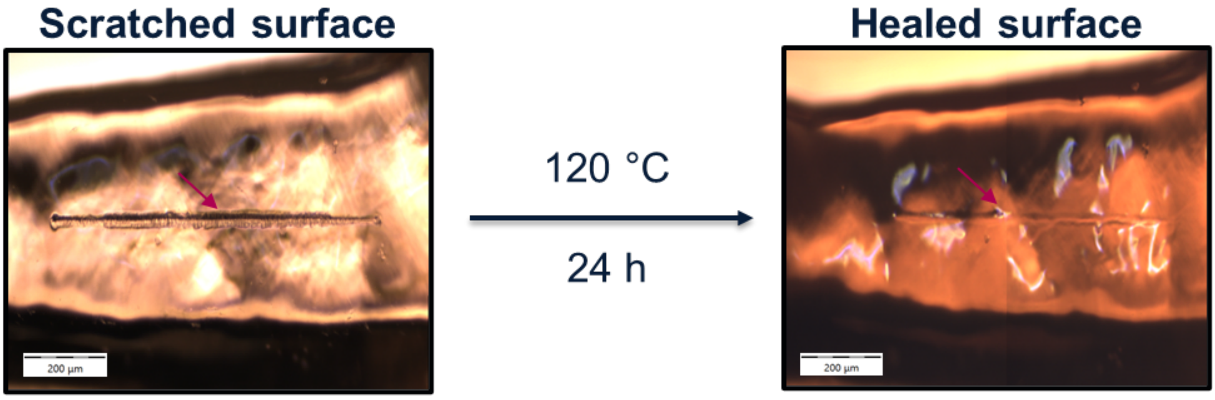

Figure S23: Optical images of the scratch on the surface introduced with a normal force of 100 mN (left) and the healed scratch after 24 h at 120 °C (right) for metallopolymer **MP2c**.

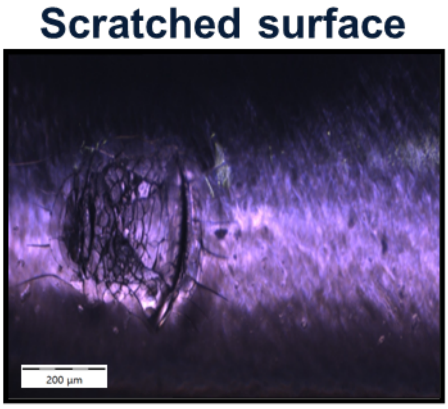

Figure S24: Optical image of the scratch on the surface introduced with a normal force of 100 mN for metallopolymer **MP3c**.

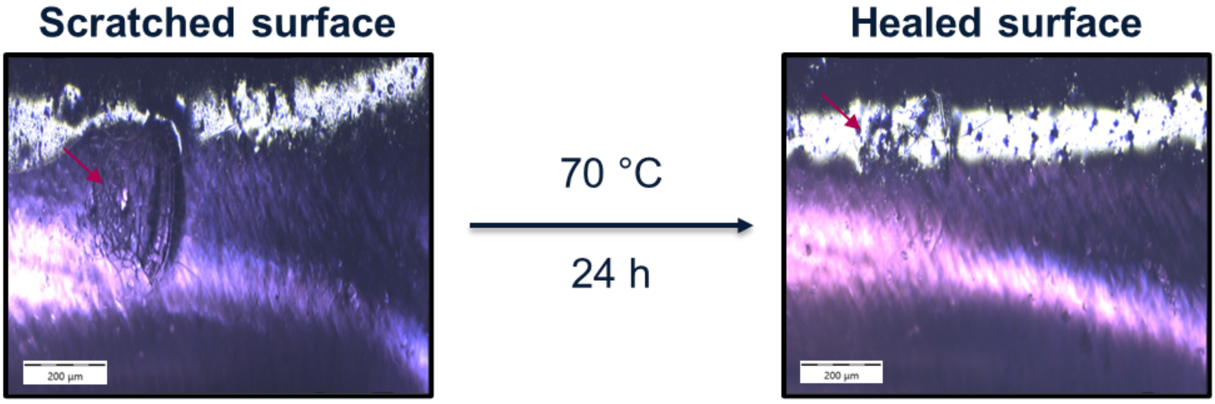

Figure S25: Optical images of the scratch on the surface introduced with a normal force of 20 mN (left) and the healed scratch after 24 h at 70 °C (right) for metallopolymer **MP3c**.

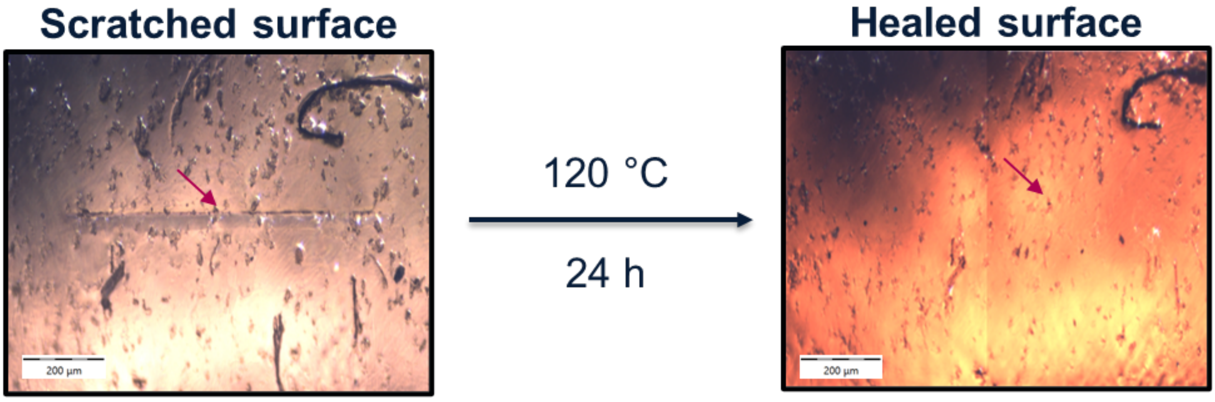

Figure S26: Optical images of the scratch on the surface introduced with a normal force of 100 mN (left) and the healed scratch after 24 h at 120 °C (right) for metallopolymer **MP4c**.

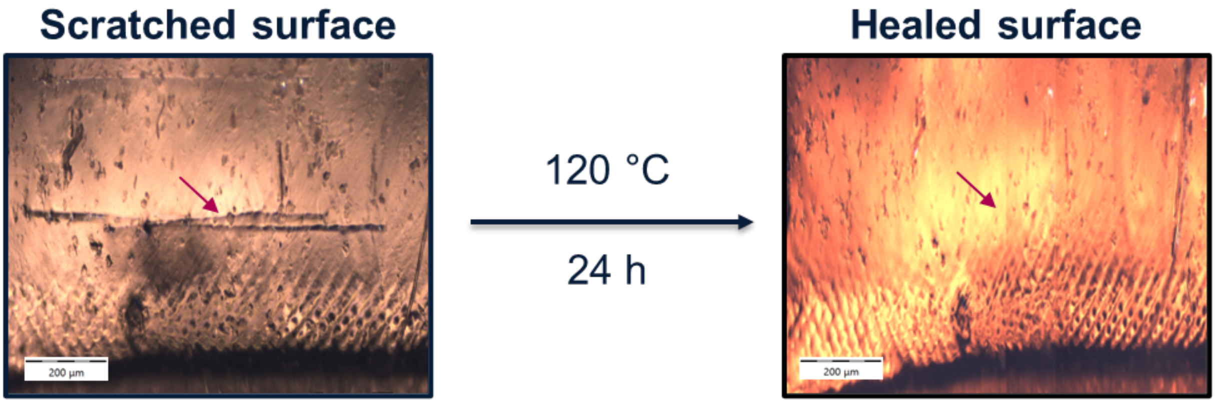

Figure S27: Optical image of the scratch on the surface introduced with a normal force of 1000 mN (left) and the healed surface after 24 h at 120 °C (right) for the metallopolymer **MP4c**.

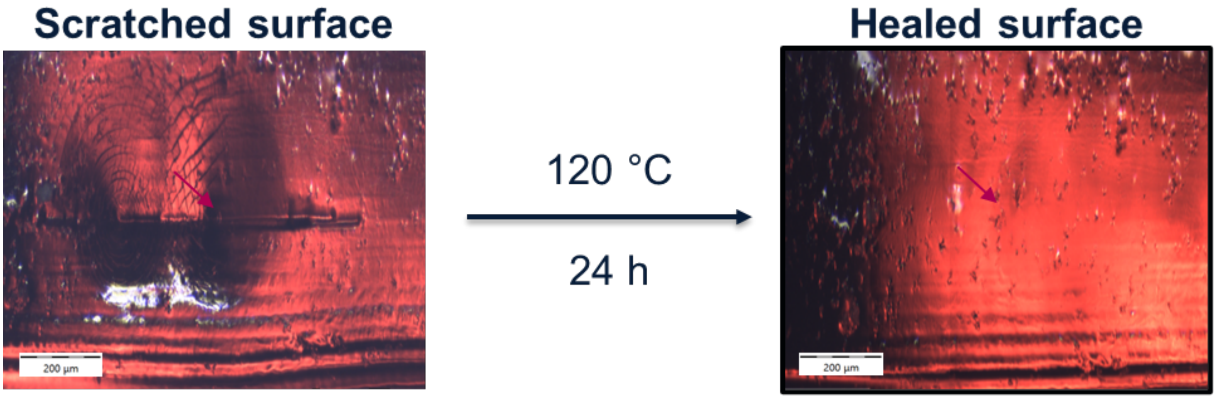

Figure S28: Optical images of the scratch on the surface introduced with a normal force of 1500 mN (left) and the healed scratch after 24 h at 120 °C (right) for metallopolymer **MP4c**.

References

[1] G. R. Fulmer, A. J. M. Miller, N. H. Sherden, H. E. Gottlieb, A. Nudelman, B. M. Stoltz, J. E. Bercaw, K. I. Goldberg, *Organometallics* **2010**, *29*, 2176-2179.
[2] J. Meurer, J. Hniopek, J. Ahner, M. Schmitt, J. Popp, S. Zechel, K. Peneva, M. D. Hager, *Beilstein J. Org. Chem.* **2021**, *17*, 2496-2504.
[3] M. Enke, F. Jehle, S. Bode, J. Vitz, M. J. Harrington, M. D. Hager, U. S. Schubert, *Macromol. Chem. Phys.* **2017**, *218*, 1600458.
[4] M. Klein, M. F. Agyemang, O. Müschke, M. Schmitt, J. Popp, M. D. Hager, S. Zechel, U. S. Schubert, *Virtual Phys. Prototyp.*, submitted.
